# Microbial Mineral Gel Network for Enhancing the Performance of Recycled Concrete: A Review

**DOI:** 10.3390/gels11080581

**Published:** 2025-07-27

**Authors:** Yuanxun Zheng, Liwei Wang, Hongyin Xu, Tianhang Zhang, Peng Zhang, Menglong Qi

**Affiliations:** 1 School of Water Conservancy and Transportation, Zhengzhou University, Zhengzhou 450001, China; yxzheng@zzu.edu.cn (Y.Z.); wangliwei@gs.zzu.edu.cn (L.W.); zhangpeng@zzu.edu.cn (P.Z.); qimenglong@stu.zzu.edu.cn (M.Q.); 2 Yellow River Laboratory, Zhengzhou University, Zhengzhou 450001, China

**Keywords:** recycled concrete, microbial mineral gel, mechanical properties, crack self-healing

## Abstract

The dramatic increase in urban construction waste poses severe environmental challenges. Utilizing waste concrete to produce recycled aggregates (RA) for manufacturing recycled concrete (RC) represents an effective strategy for resource utilization. However, inherent defects in RA, such as high porosity, microcracks, and adherent old mortar layers, lead to significant performance degradation of the resulting RC, limiting its widespread application. Traditional methods for enhancing RA often suffer from limitations, including high energy consumption, increased costs, or the introduction of new pollutants. MICP offers an innovative approach for enhancing RC performance. This technique employs the metabolic activity of specific microorganisms to induce the formation of a three-dimensionally interwoven calcium carbonate gel network within the pores and on the surface of RA. This gel network can improve the inherent defects of RA, thereby enhancing the performance of RC. Compared to conventional techniques, this approach demonstrates significant environmental benefits and enhances concrete compressive strength by 5–30%. Furthermore, embedding mineralizing microbial spores within the pores of RA enables the production of self-healing RC. This review systematically explores recent research advances in microbial mineral gel network for improving RC performance. It begins by delineating the fundamental mechanisms underlying microbial mineralization, detailing the key biochemical reactions driving the formation of calcium carbonate (CaCO_3_) gel, and introducing the common types of microorganisms involved. Subsequently, it critically discusses the key environmental factors influencing the effectiveness of MICP treatment on RA and strategies for their optimization. The analysis focuses on the enhancement of critical mechanical properties of RC achieved through MICP treatment, elucidating the underlying strengthening mechanisms at the microscale. Furthermore, the review synthesizes findings on the self-healing efficiency of MICP-based RC, including such metrics as crack width healing ratio, permeability recovery, and restoration of mechanical properties. Key factors influencing self-healing effectiveness are also discussed. Finally, building upon the current research landscape, the review provides perspectives on future research directions for advancing microbial mineralization gel techniques to enhance RC performance, offering a theoretical reference for translating this technology into practical engineering applications.

## 1. Introduction

As a globally prevalent construction material, concrete production has surpassed 2.36 billion tons annually [1]. This extensive use of concrete necessitates the consumption of vast quantities of natural aggregate (NA) while simultaneously generating significant greenhouse gas emissions. Statistics indicate that approximately 10% of anthropogenic carbon dioxide emissions are attributable to the production and transportation of concrete [2]. Concurrently, rapid urban growth is exacerbating the environmental impact of construction waste in cities, making effective waste management a critical challenge for modern cities. Utilizing waste concrete from construction debris to produce recycled aggregate (RA) through crushing, screening, and washing for recycled concrete (RC) manufacturing offers a dual benefit, reducing the demand for NA and facilitating the recycling and utilization of building waste [3]. RA, however, inherently exhibits enhanced porosity and reduced strength. This is primarily attributed to the presence of adhered old mortar, which negatively affects the performance of RC and consequently impedes its broader acceptance and application [4,5]. Studies have shown that RC exhibits a reduction in slump and slump fluidity of 32.3% and 31%, respectively, compared to natural aggregate concrete (NAC) [6] with generally lower flow and consistency values [7]. Verian et al. [8] reported that RC mixtures require an additional 5–15% water content to achieve comparable workability to NAC. Furthermore, replacing NAC with RA can result in a compressive strength reduction of up to 27% [9].

To enhance the engineering properties of RA, various modification methodologies have been developed, broadly categorized into two main groups, namely (1) removal of surface-adherent mortar and (2) strengthening of surface-adherent mortar. The removal of adherent mortar (AM) is often achieved through mechanical grinding. While this process can remove a significant amount of AM, it can also induce microcracks on the RA surface, potentially weakening its mechanical strength and affecting long-term durability [10]. Acid treatment methodologies offer effective dissolution of cementitious hydration products adhering to RA surfaces. However, while old AM is removed, the CO_2_ produced by the reaction poses environmental pollution concerns and may increase the presence of harmful ions, such as chloride ions [11,12,13]. Regarding the strengthening of surface-adherent mortar, carbonation treatment can effectively improve the surface compactness and compressive strength of RA while reducing water absorption. Nevertheless, it is crucial to consider the natural carbonation of RA that occurs during long-term use and its potential for performance deterioration [14,15]. Other approaches to reducing water absorption include soaking in a polyvinyl alcohol emulsion [16] and applying a volcanic ash slurry [17]; however, the interfacial compatibility between polymers and cementitious materials remains a key constraint to their broader application. Treatment of RA with a sodium silicate solution is another option, but it may initiate an alkali–silica reaction, potentially affecting the long-term durability of the concrete [18].

**Figure 1:**
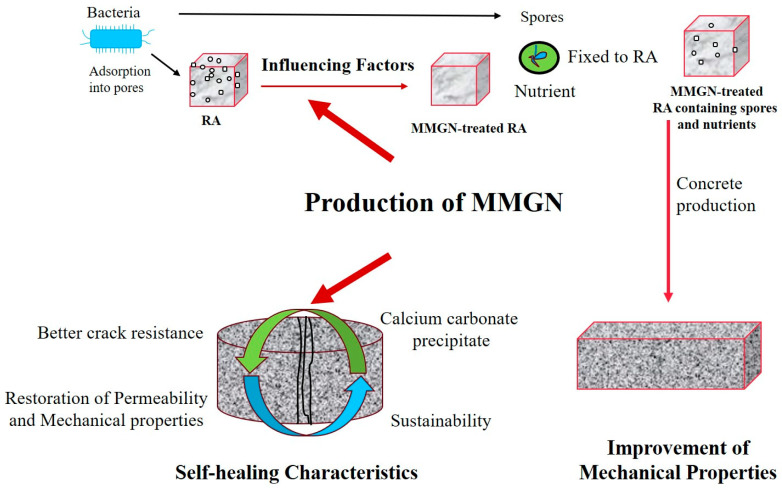
Conceptual figure illustrating MMGN formation and action in RC.

Microbially induced carbonate precipitation (MICP) technology presents a promising avenue for addressing performance limitations associated with RC [19]. This technique leverages the metabolic activity of mineralizing microorganisms to decompose substrates, resulting in the generation of carbonate ions (CO_3_^2−^). Subsequently, in the presence of an external supply of free calcium ions (Ca^2+^), these ions combine within the surface pores and microcracks of RA, promoting the formation of nano-sized CaCO_3_ crystals. These crystals are attracted and directionally linked through intermolecular forces, ultimately resulting in the formation of a three-dimensional interwoven calcium carbonate gel network within the pores and on the surface of the RA [20]. This microbial mineral gel network (MMGN) effectively fills the pores of the RA, thereby reducing its water absorption while simultaneously enhancing the aggregate’s stiffness and intrinsic strength [21]. More importantly, the biomineralized gel layer formed on the surface of the RA acts to strengthen the interfacial transition zone (ITZ) between the aggregate and the cementitious matrix, consequently improving the physical and mechanical properties of the resulting RC [22,23,24]. An even more innovative strategy involves the pre-embedding of mineralizing microbial spores into the pores of the RA during concrete mixing, enabling the production of self-healing RC. Following crack formation in the concrete, environmental conditions (such as ingress of moisture and air) activate these spores within the RA pores. The activated bacteria initiate metabolic processes within the cracks, inducing the precipitation of calcium carbonate crystals. Crucially, these crystals undergo ordered self-assembly via intermolecular forces, forming a gel-like substance characterized by a three-dimensional network structure. This gel serves a dual function, remediating the crack by sealing it and providing partial structural support. A conceptual figure illustrating MMGN formation and action in RC is presented in Figure 1. Compared to conventional RA enhancement techniques, MICP technology presents distinct advantages, including greater environmental friendliness, improved material compatibility, and crucially, the ability to impart self-diagnostic and self-healing properties to RC [25,26]. These attributes significantly reduce the long-term maintenance costs of concrete structures. Consequently, the strategy of utilizing microbial mineral gel networks to enhance RC technology is attracting increasing research attention.

Owing to the unique merits of MMGN for performance enhancement in RC, this paper reviews the use of microorganisms to produce calcium carbonate gel networks to reinforce RA, thereby enhancing RC, as well as the incorporation of bacterial spores into RA to impart self-healing properties to RC, thereby providing a theoretical foundation for subsequent technological refinement and engineering applications. The factors influencing the effectiveness of microbial mineralization in strengthening RA are systematically analyzed, encompassing the specific bacterial types and concentrations employed, alongside relevant environmental parameters, including temperature, pH, and concentrations of calcium ions and urea; optimization strategies for the selection of each factor are subsequently discussed. The enhancement effects of MICP technology on the key mechanical properties of RC are then critically examined, with the mechanisms underlying the microstructural improvements imparted by the microbial mineral gel network further elucidated. Research findings concerning the self-healing efficiency of self-healing RC are subsequently summarized, including such metrics as crack width healing ratio, permeability recovery, and mechanical property restoration; the key factors affecting the self-healing performance are also discussed. Finally, future research directions for advancing recycled concrete technology through microbial mineral gel networks are presented.

## 2. Factors Influencing RA Enhancement by MMGN

The inherent porosity of RA, stemming from the presence of residual mortar on its surface, presents a potential avenue for MICP. Bacteria capable of facilitating MICP may readily adsorb the pores of the RA, forming a microbial mineral gel network. This network preferentially fills cracks and pores, thereby serving a protective and filling function. Additionally, the gel forms chemical bonds (Ca-O-Si) with calcium silicate hydrate (C-S-H), significantly enhancing interface density, and thereby improving the overall performance of the RA [27]. This chapter will first characterize the microbial communities associated with RA treatment and their mineralization pathways. Subsequently, the effects of parameters, such as bacterial concentration, culture temperature, pH conditions, calcium ion availability, and urea decomposition rate, on the aggregate performance enhancement are rigorously evaluated.

### 2.1. Bacteria Applied to Enhance RA

#### 2.1.1. Bacterial Species

(1) Ureolytic bacteria

**Figure 2:**
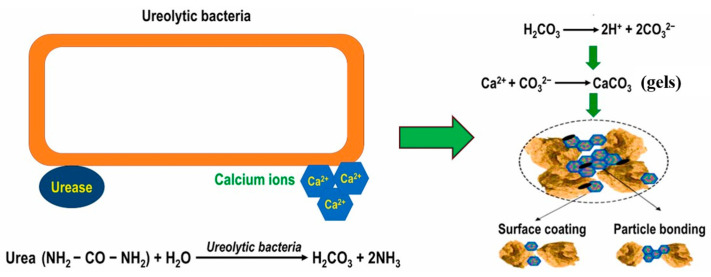
Mechanism of CaCO_3_ gels production from urea hydrolysis [28].

Ureolytic bacteria represent the most frequently employed bacterial group in concrete applications. Figure 2 illustrates the precipitation process facilitated by these bacteria [27,28]. Ureolytic bacteria, including species, such as *Bacillus megaterium* [29] and *Sporoscarcina pasteurii* [30], induce CaCO_3_ gels by secreting urease. Urea diffuses into the bacterial cell down a concentration gradient, and the urease secreted by these bacteria catalyzes urea hydrolysis, yielding ammonium and carbonate ions. The generated carbonate then reacts with calcium ions present in the surrounding environment, leading to the formation of CaCO_3_ gel precipitates [31]. The resulting gel can both coat the RA surface and serve as a binding agent between particles, thereby contributing to its strengthening and repair.

(2) Denitrifying bacteria

Denitrifying bacteria, capable of growth and mineralization in oxygen-deprived environments, represent a significant class of anaerobic bacteria. For instance, Liu et al. (2021) successfully employed the denitrifying bacterium *Pseudomonas* sp. (American Type Culture Collection, No. ATCC13867) to enhance RA [32]. The mineralization process of denitrifying bacteria, as detailed in Equations (1)–(5) [33,34], involves a biochemical pathway where nitrate ions are reduced to nitrogen gas, which is then released into the atmosphere. Simultaneously, the generated carbon dioxide is converted into carbonate ions within the alkaline environment, facilitating their combination with calcium ions to precipitate CaCO_3_ gels. The remaining byproducts, namely nitrogen and water, are naturally occurring and readily utilized substances. This approach circumvents the production of ammonia and eliminates the need for urea addition, potentially offering improved economic and environmental advantages compared to other MICP methods for aggregate treatment.

(1) $$2HCOO^{-}+2NO_{3}^{-}+2H^{+}\to 2CO_{2}+2H_{2}0+2NO_{2}$$

(2) $$HCOO^{-}+2NO_{2}^{-}+3H^{+}\to CO_{2}+2H_{2}0+2NO$$

(3) $$HCOO^{-}+2NO+H^{+}\to CO_{2}+H_{2}0+2N_{2}O$$

(4) $$HCOO^{-}+N_{2}O+H^{+}\to CO_{2}+2H_{2}0+N_{2}$$

(5) $$CO_{2}+Ca^{2+}+OH^{-}\to CaCO_{3}+H_{2}0$$

While denitrifying bacteria offer a promising avenue for biomineralization, their anaerobic nature presents a significant limitation in oxygen-rich environments. Furthermore, the relatively slow metabolic activity of anaerobic bacteria, in general, translates to a lower rate of CaCO_3_ precipitation compared to ureolytic pathways. This slower precipitation often necessitates extended treatment durations. Consequently, denitrifying bacteria are currently less frequently employed in RA consolidation efforts. Further research and optimization are, therefore, essential to enhance their performance and broaden their applicability in practical field settings.

(3) Organic compound-converting bacteria

**Figure 3:**
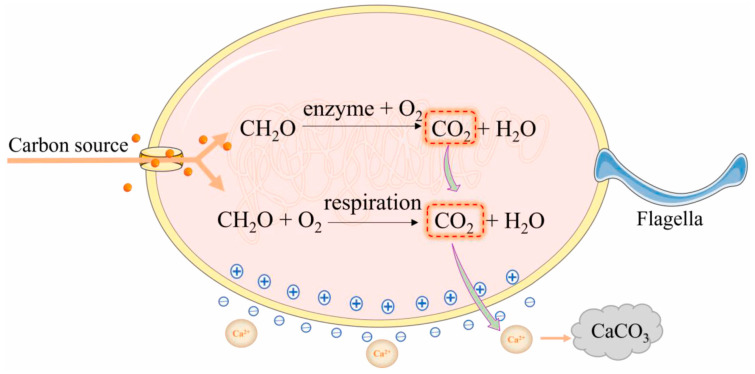
Calcium carbonate production by organic transforming bacteria [36].

Organic compound-converting bacteria enhance RAs through a metabolism-driven process where organic matter serves as a carbon source, resulting in the formation of CaCO_3_ gels as a biomineralization byproduct. Current applications of these bacteria in reinforcing RAs include strains of *B. cohnii* [22], *Bacillus subtilis* [19,35], and *B. pseudofirmus* [7]. As illustrated in Figure 3, the biomineralization process of these bacteria involves the absorption and decomposition of organic carbon sources into carbon dioxide and water, a process typically regulated by enzymatic activity. The generated carbon dioxide readily converts to carbonate ions, which subsequently precipitate with calcium ions, leading to the formation of CaCO_3_ gels. These bacteria can utilize a variety of organic compounds to facilitate precipitation, including C_6_H_10_CaO_6_, Ca(C_2_H_3_O_2_)_2_, Ca(NO_3_)_2_, and CaCl_2_.

Notably, the nutrients utilized by these organic compound-converting bacteria are commonly employed in engineering applications, rendering them suitable for practical implementation. However, it is crucial to acknowledge that this biomineralization process requires oxygen for CaCO_3_ gel production; consequently, low-oxygen environments can lead to reduced precipitation rates, thus potentially limiting the overall rate of mineralization.

(4) Carbon-fixing bacteria

**Figure 4:**
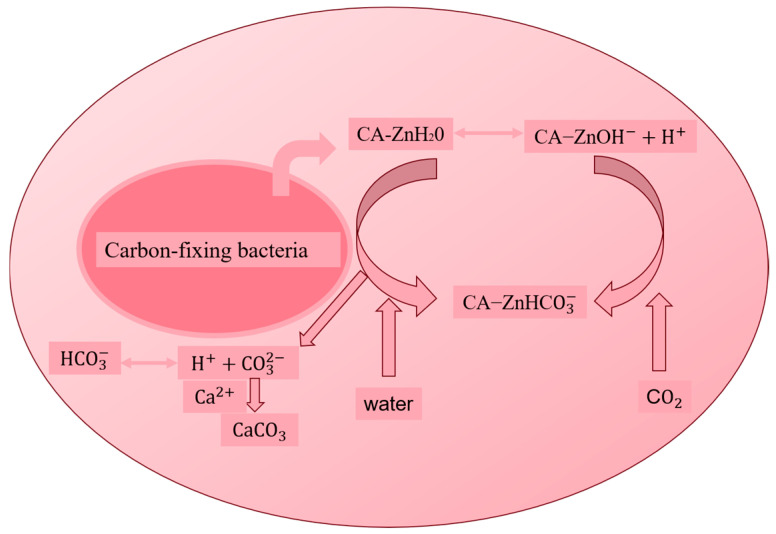
Calcium carbonate production by carbon-fixing bacteria.

Zinc-dependent enzymes biologically catalyze the hydration of CO_2_ to carbonic acid. The subsequent environmental dissociation of carbonic acid yields carbonate ions, which can then form solid gels with Ca^2+^. Carbon-fixing bacteria, such as *Bacillus mucilaginosus* [37] and Bacillus cereus [38], are frequently employed by researchers in this context (Figure 4 illustrates the reaction flow of carbon-fixing bacteria). At the active site of these enzymes, zinc ions (Zn^2+^) establish a tetrahedral coordination structure, typically involving three histidine residues and a water molecule. At physiological pH, this water molecule dissociates to form a hydroxyl group (OH^−^), which then undergoes nucleophilic attack on CO_2_ [39]. The resulting ionization produces carbonate ions that combine with artificially added calcium ions, leading to the formation of CaCO_3_ gels.

Carbonic anhydrase from carbon-fixing bacteria exhibits a high catalytic rate, reported to reach up to 440,000 cycles per second [40]. This reaction proceeds under ambient temperature and pressure conditions, catalyzing the production of high-purity calcite. The process additionally facilitates carbon dioxide sequestration, thereby contributing to the mitigation of carbon emissions. However, the activity of these bacterial strains is strictly constrained by pH (7.5–9.5) and temperature (25–35 °C), necessitating further investigation to optimize their performance under a broader range of environmental conditions.

(5) Fungi

While fungi possess the capacity to form hydrophobic biofilm layers that repel water [41], their application in construction faces certain limitations. Compared to bacterial biofilms, fungal biofilms exhibit enhanced durability and are more effectively integrated into RA due to the presence of chitin in their cell walls [42]. This chitin facilitates the adsorption of calcium ions to the cell wall surface, creating microenvironments conducive to CaCO_3_ gels [43]. Recent research has explored the potential of fungi, such as *Fusarium oxysporum* and *Trichoderma longibrachiatum*, in the self-healing of reinforced concrete [44]. For instance, the incorporation of fungal-cemented RA has demonstrated the ability to heal cracks up to 1.34 mm. However, the potential biosafety concerns associated with the direct use of fungal spores currently constrain their widespread application in the construction industry.

(6) Other pathways

Enzyme-induced carbonate precipitation (EICP) offers another promising approach to enhance RA by producing CaCO_3_ gels. The mechanism of EICP is to use urease extracted from agriculture, microorganisms, and fungi as a catalyst to decompose urea into CO_3_^2−^, thereby generating calcium carbonate precipitation [45,46]. Because EICP technology lacks microorganisms, it cannot embed germinable bacterial spores in concrete, so EICP cannot provide self-healing capability to RC [47,48]. However, they can all be used to enhance RA and represent innovative and increasingly popular enhancement methods.

#### 2.1.2. Bacterial Concentration

**Figure 5:**
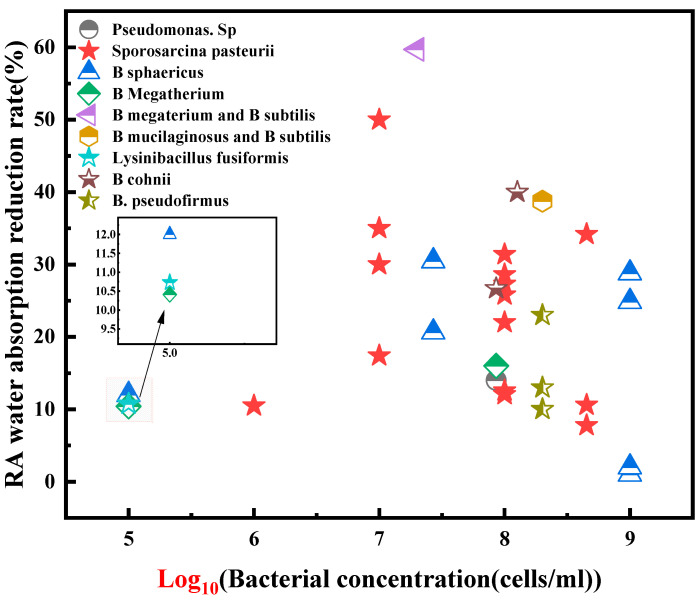
The decreasing rate of water absorption of RA under different bacteria and concentrations. [19,21,24,30,32,35,37,49,50,51,52,53,54,55].

Bacterial concentration plays a significant role in enhancing the property of RA. Generally, an elevated bacterial concentration enhances the modification effect. For instance, Qiu et al. [49] demonstrated that increasing the bacterial concentration from 10^6^ to 10^8^ cells/mL led to a progressive reduction in the water absorption capacity of RA, ranging from 8% to 16%. This effect is attributed to the increased bacterial population facilitating more efficient urea hydrolysis, thereby elevating CaCO_3_ gels availability and providing a greater number of nucleation sites for CaCO_3_ gels network, ultimately improving precipitation yields. However, excessively high bacterial concentrations can be detrimental, potentially exceeding the available surface area of the RA, thereby limiting the efficiency of CaCO_3_ gels. Feng et al. [35], in their study using a consortium of two bacterial species to enhance RA, observed an optimal mineralization rate of 80.56% at a total bacterial concentration of 2 × 10^8^ cells/mL, while a higher concentration of 10^9^ cells/mL resulted in a comparatively lower mineralization rate. Consequently, many studies have adopted a bacterial concentration of approximately 10^8^ cells/mL to achieve enhanced effectiveness in RA bio-treatment. As illustrated in Figure 5, the water absorption reduction percentages of RA treated with various bacterial species at concentrations near 10^8^ cells/mL typically range from 10% to 40%. Furthermore, synergistic effects have been observed in dual-species treatments, with water absorption reductions reaching 59.7% and 38.7%, indicating superior performance compared to single-species applications [35,50].

### 2.2. Environmental Factors

Microbial mineralization is a biochemical reaction, and environmental factors can significantly influence the formation of MMGN. Therefore, this section provides a detailed summary of the effects of various environmental factors on MICP-enhanced RA.

#### 2.2.1. Temperature

Temperature, a critical environmental parameter, exerts a dual regulatory effect on the MICP process, influencing both bacterial metabolic activity and the CaCO_3_ gels’ deposition. Research indicates that common mineralization microbes exhibit peak biological activity and achieve their fastest CaCO_3_ precipitation rates within the temperature range of 20–37 °C [56,57]. Within this optimal thermal window, elevated temperatures accelerate urea hydrolysis and enhance molecular interaction kinetics [58]. For example, the bacterial decomposition of urea proceeds more rapidly at 35 °C than at 25 °C, resulting in a greater quantity of precipitate [45]. Furthermore, urease activity remains stable at 35 °C [59], while experiencing a 55% decrease at 47 °C [60], and effectively ceasing below 5 °C. Biological studies of denitrification and urea hydrolysis [61] indicate peak reaction rates at 30 °C, with fungal proliferation optimized around 29 °C. Therefore, precise temperature control is essential for optimizing the efficiency and stability of the MICP process.

#### 2.2.2. pH

The influence of pH on bacterial survival and activity is multifaceted, and impacts carbonate gels’ stability [62,63]. Many bacteria employed in MICP favor alkaline conditions, and the urea hydrolysis process itself generates ammonium and bicarbonate ions, thereby elevating the surrounding pH [64] and promoting production of CaCO_3_ gels. The rate of bacterial urea decomposition is demonstrably pH-dependent. For instance, *S. pasteurii* exhibits a ten-fold increase in precipitation at pH 9 compared to pH 7 [49]. Most denitrifying bacteria exhibit optimal activity within a neutral to slightly alkaline pH range (7.0–8.0) [32]. Feng et al. [35] studied the effect of pH on the mineralization rate of the organic transforming bacteria *Bacillus subtilis* and carbon-fixing bacteria *Bacillus mucilaginosus*, and pointed out the organic transformer *Bacillus subtilis* was more sensitive to pH changes than the carbon-fixing bacterium *Bacillus mucilaginosus*. Secondly, pH levels critically impact CaCO_3_ crystal morphology by governing direction-specific growth rates. Simultaneously, pH-induced changes in bacterial activity accelerate or decelerate precipitation kinetics, thereby controlling the structural evolution of CaCO_3_ gels [52].

#### 2.2.3. Calcium Ions

**Figure 6:**
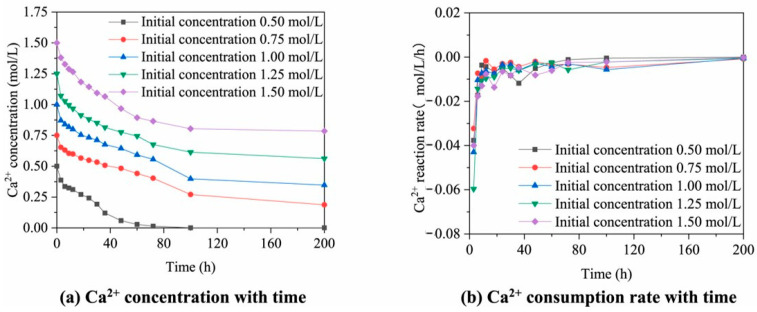
Changes in calcium ion concentration during the MICP process [21].

**Figure 7:**
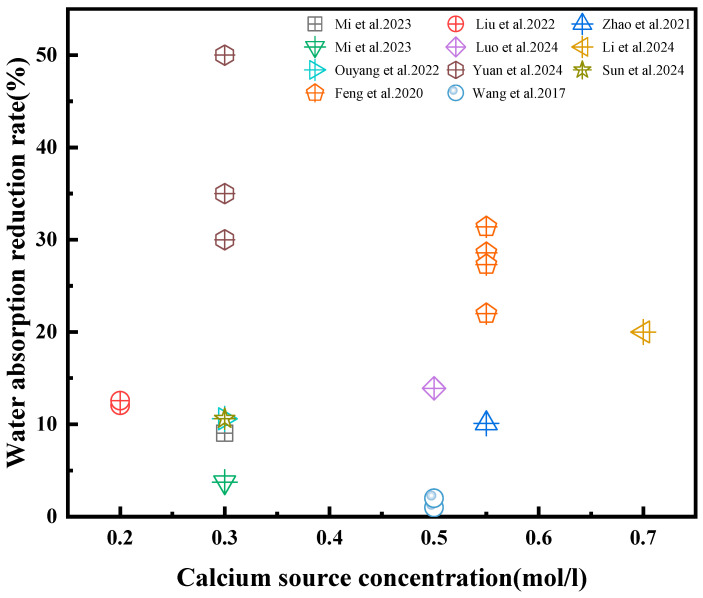
Calcium ion concentrations used to enhance RA in various studies. [19,24,30,45,51,52,53,54,55,64,65].

Calcium ions are crucial in microbial mineralization for enhancing RA, with their concentration directly impacting bacterial reaction rate. As illustrated in Figure 6a,b, calcium ion concentration typically exhibits a gradual decline over time during the MICP process. Higher initial substrate concentrations correlate with greater residual calcium ions, potentially indicating that excessive calcium levels can inhibit bacterial enzyme activity [21]. Conversely, elevated calcium ion concentrations also promote increased CaCO_3_ precipitation, potentially mitigating the impact of slower urea decomposition rates. Feng et al. [53] demonstrated that increasing calcium ion concentrations from 0.05 to 0.55 mol/L resulted in a corresponding increase in CaCO_3_ gels yields from 0.464% to 1.901%. Figure 7 further highlights the range of calcium ion concentrations (typically 0.3–0.6 mol/L) employed for enhancing RA in various experimental studies. The variability in optimal concentrations across these studies likely arises from differences in bacterial species, urea availability, temperature, and pH conditions. Therefore, the selection of an appropriate calcium ion concentration necessitates case-specific validation under representative environmental conditions.

#### 2.2.4. Urea

**Figure 8:**
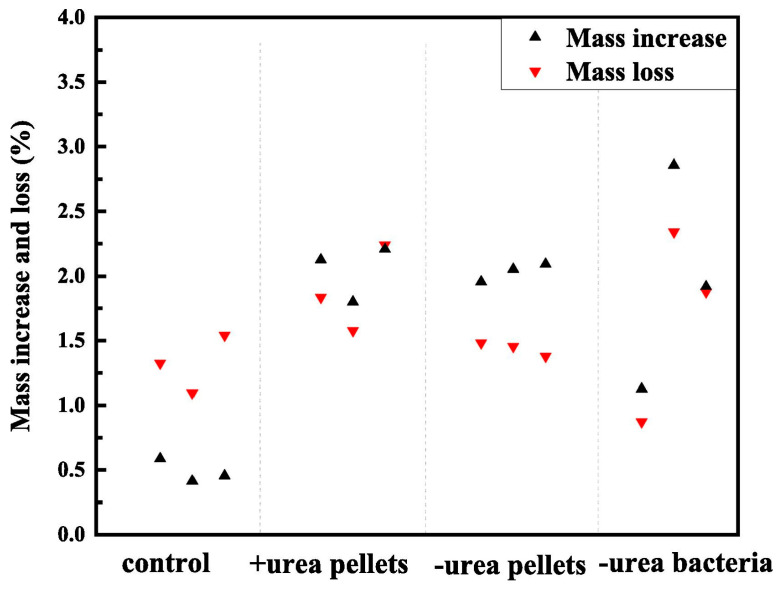
The increase in quality after using different bacterial solutions for biological sedimentation treatment of RA and the loss of quality after ultrasonic attack [69].

Urea is essential for CaCO_3_ gel production by urea-hydrolyzing bacteria; thus, its concentration must be carefully controlled. Research indicates an inverse relationship between urea concentration and precipitate particle size; lower generation rates result in smaller particles, promoting increased particle contact and enhanced bond strength [66]. Excessively high urea concentrations, conversely, can lead to rapid CaCO_3_ formation primarily at the surface, occluding pore openings and crack entrances without effectively reinforcing deeper layers of the RA [67,68]. Furthermore, the inducible nature of bacterial urease, whose production is directly related to urea availability [69,70], affects the modification effect on RA. For instance, using bacterial solutions cultured with or without substrate urea for biological treatment of RA, a comparative analysis was conducted on the precipitation quality of RA and the mass loss after ultrasonic treatment (Figure 8). Experimental results indicated similar mass increases across all test groups, with 2.04% for the +urea pellets group, followed by the -urea pellets and -urea bacteria groups with averages of 2.03% and 1.97%, respectively. This suggests the limited influence of bacterial solution types on total precipitate mass. However, the -urea bacteria and -urea pellets groups demonstrated superior cohesive strength of deposits, exhibiting mass losses of merely 1.70% and 1.44% during ultrasonication, respectively, compared to 1.88% for the +urea pellets group. These findings suggest that bacterial mineralization without substrate urea cultivation may result in slower precipitation rates but generates more viscous carbonate deposits. This gradual precipitation process facilitates more effective deposition of CaCO_3_ gels within RA defects, thereby achieving significantly enhanced pore-filling and microcrack remediation capabilities.

## 3. Mechanical Properties of MMGN-Enhanced RC

**Figure 9:**
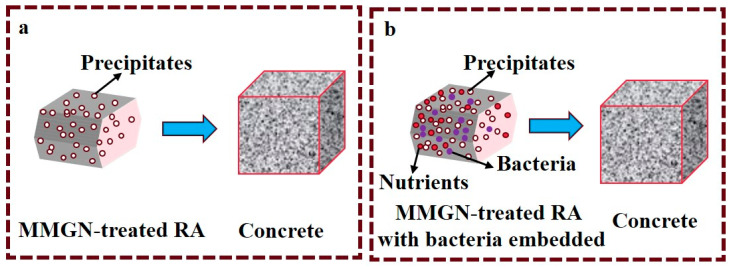
Two types of MMGN-enhanced RC. (**a**) Concrete made with MMGN enhanced RA; (**b**) Self-healing Concrete made by using MMGN enhanced RA to immobilize bacterial spores.

There are two types of MMGN-enhanced RC. The first involves formulating RC enhanced solely with MMGN-treated RA, as illustrated in Figure 9a. The second entails adsorbing bacterial spores with MMGN-treated RA, followed by the addition of urea and nutrients during concrete mixing to facilitate bacterial growth and enable self-repairing capabilities, as depicted in Figure 9b. Consequently, this section primarily focuses on analyzing the strength variations and the interfacial reinforcement mechanisms associated with these two types of concrete.

### 3.1. Compressive Strength

**Figure 10:**
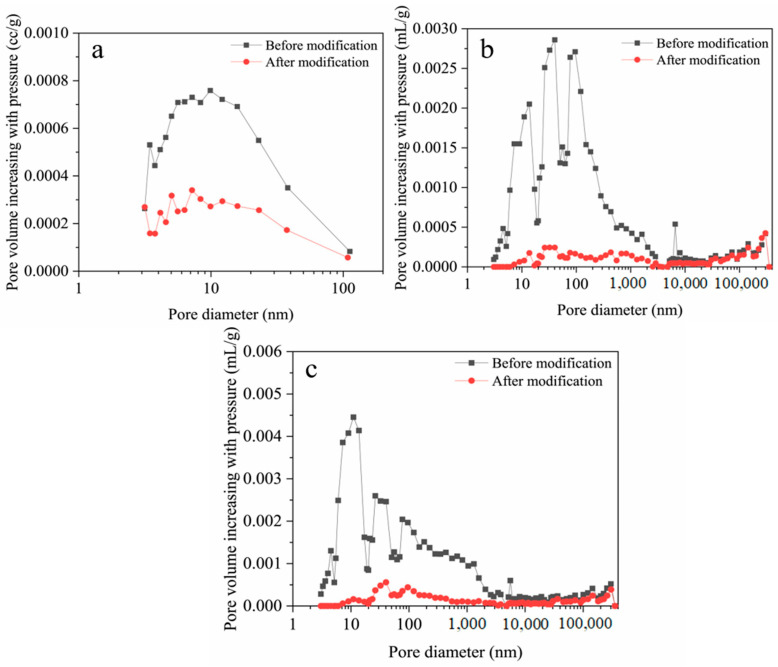
Porosity change in RA after MICP treatment: (**a**) RCA; (**b**) RMA; (**c**) RFA [21].

The bacteria-induced gels’ precipitation process fosters a stronger interfacial bond between RA and the cement paste, resulting in tangible structural improvements. Concurrently, the biogenic precipitates infill microcracks within the RA matrix, leading to a denser internal structure and consequently enhanced compressive strength [71]. Tang et al. [21] demonstrated the efficacy of MICP in altering RA porosity, as illustrated in Figure 10. This figure presents a comparative analysis of porosity in three RA types—recycled coarse aggregate (RCA), recycled medium aggregate (RMA), and recycled fine aggregate (RFA)—before and after MICP treatment. Following MICP modification, the finer pores within the coarse and medium aggregates were effectively filled, while the pore volume of the fine aggregates was significantly reduced. These alterations fundamentally modify the inherent properties of the RA, suggesting that concrete incorporating these treated RAs would exhibit further improvements in strength.

**Figure 11:**
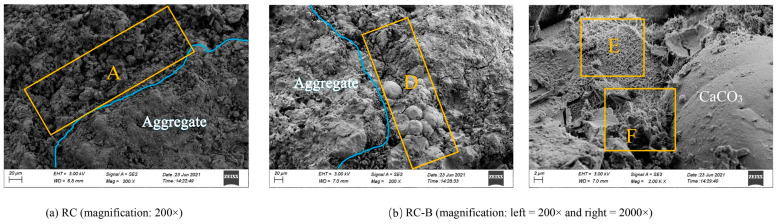
SEM images of RC: (**a**) Concrete made with untreated RCA (RC); (**b**) concrete made with RCA treated with MICP (RC-B) [10].

The incorporation of MMGN-treated RCA has been shown to effectively enhance the compressive strength of concrete. This improvement is attributed to two primary mechanisms, namely the deposition of CaCO_3_ gels within the pores and on the surfaces of the RCA, and the role of this generated CaCO_3_ gels as nucleation sites, accelerating cement hydration [72], ultimately leading to a higher compressive strength of RC. Research has reported increases in the cubic compressive strength of RC by as much as 19% [65]. Furthermore, the ratio of bacterial solution to urea solution has been identified as a critical factor influencing compressive strength. Optimal results, with a 19.54% increase in compressive strength after 28 days compared to unmodified RCA, were achieved with a bacterial solution to urea solution ratio of 5:6 [24]. The beneficial impact of MMGN-treated RCA on compressive strength is further substantiated by scanning electron microscopy (SEM) [10]. As illustrated in Figure 11a, the aggregate surface in Zone A of concrete (RC) prepared with untreated RCA exhibits residual old mortar, which compromises the ITZ performance. In contrast, Figure 11b reveals the ITZ of microbial-treated RCA concrete (RC-B), where Zone D demonstrates spherical crystals embedded within the cementitious matrix. The transformation of C-S-H from a planar configuration (Zone E) to a three-dimensional spatial network (Zone F) significantly enhances the mechanical properties within the ITZ. The enhancement of mechanical properties through MICP can be attributed to the effective filling and repair of microcracks and large voids in RA by the obtained gel precipitates, thus improving its inherent weaknesses. In addition, the good compatibility of CaCO_3_ with the newly formed mortar gel enhances the compactness of the ITZ. Crucially, the presence of CaCO_3_ gel promotes the transformation of C-S-H and forms new chemical bonds, which, in turn, strengthens the microstructure and overall integrity of the newly developed ITZ.

**Figure 12:**
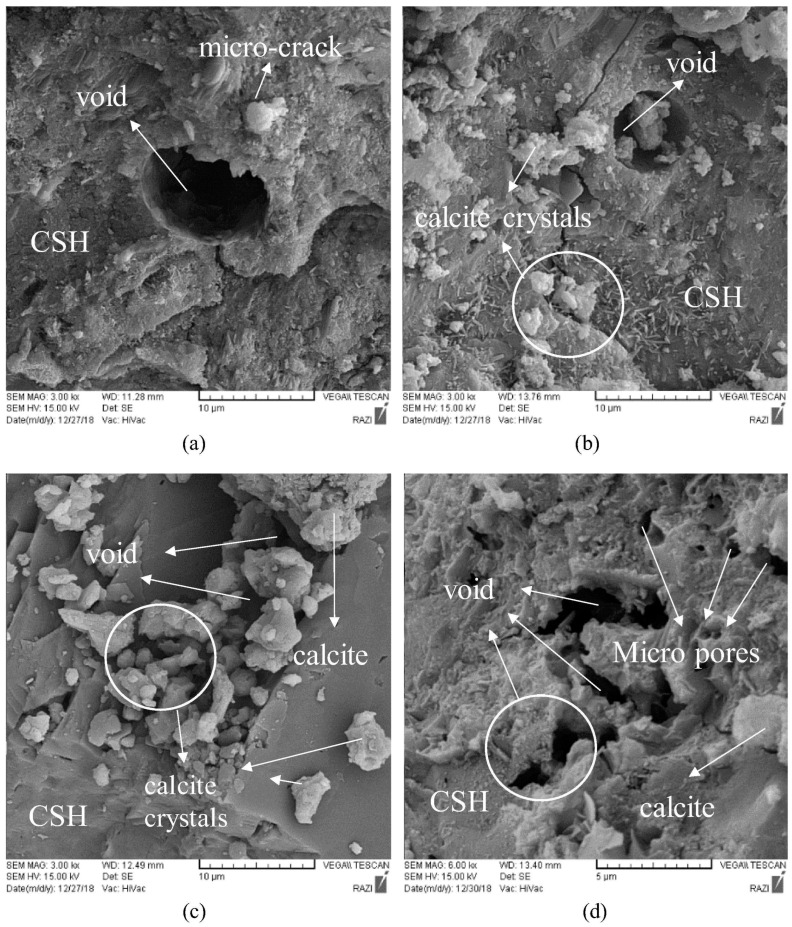
SEM images of MMGN-treated self-healing RC with different contents of calcium lactate. (**a**) Control, (**b**) 1% calcium lactate, (**c**) 3% calcium lactate, and (**d**) 5% calcium lactate [77].

However, the introduction of urea, calcium sources, and bacterial spores immobilized on RA into concrete presents potential drawbacks regarding its long-term strength. These introduced organic materials may inhibit the complete hydration process of cement, potentially weakening the interfacial bond between the cement hydration products and the RA. Furthermore, accumulated dead bacterial cells may contribute to an increased pore volume and overall porosity within the concrete matrix [73,74]. Wang et al. [75] observed a 14% reduction in the compressive strength of RC incorporating bacterial spores at 28 days. This strength reduction may be attributable to increased porosity resulting from the introduction of nutrients or from excessive nutrient depletion, leading to the formation of a less dense, “fluffy” microstructure [76]. Furthermore, the addition of calcium lactate can also negatively influence the compressive strength of RC. Research indicates that the compressive strength enhancement at 28 days achieved with a 3% addition of calcium lactate decreased to 13%, compared to the 21% enhancement observed with a 1% addition. A 5% addition of calcium lactate resulted in excessive crystal precipitation, thereby increasing porosity and decreasing the 28-day compressive strength by 7% relative to the control group [77]. This phenomenon is likely due to the overproduction of CaCO_3_ gels in the presence of high calcium concentrations, which consequently increases the overall porosity of the concrete matrix. Microscopic analysis, as illustrated in Figure 12, provides further insight. The SEM image of the control sample (Figure 12a) reveals the presence of inherent pores and cracks. Figure 12b shows CaCO_3_ gels filling some of the pores and adhering to the aggregates; however, some CaCO_3_ gels remain unutilized. Figure 12c demonstrates a significant increase in these unutilized CaCO_3_ gels, and Figure 12d illustrates an increase in overall porosity, ultimately contributing to a reduction in compressive strength. In addition, while the addition of calcium nitrate and urea had no significant effect on the concrete properties, the addition of yeast extract significantly affected the mechanical properties [78].

In summary, the incorporation of RA reinforced with MICP modifies the microstructure of the resulting RC. Specifically, the newly formed ITZs exhibit an improved structure, attributable to the newly generated CaCO_3_ gels crystals promoting the transition of C-S-H, thereby enhancing compressive strength. While the compressive strength of MMGN-enhanced RC generally improves, as evidenced by a compilation of results from various studies (Table 1), some instances reveal a reduction in compressive strength, potentially due to the introduction of nutrients and bacterial spores, which may disrupt the hydration process under certain conditions.

### 3.2. Splitting Tensile Strength

The MMGN-treated RC exhibited a superior splitting tensile strength compared to its untreated counterpart, primarily attributable to enhanced bonding properties within the ITZ between the original and new mortars. The inherent characteristics of RA often lead to the generation of a greater number of microcracks during fracture, resulting in a comparatively weaker ITZ and a corresponding reduction in splitting tensile strength [81]. Supporting this observation, Amjad et al. [82] reported a 14.6–17.6% increase in the splitting tensile strength of treated recycled brick aggregate concrete relative to control specimens, further validating the beneficial effects of MICP treatment on the tensile and bond characteristics of RC [30]. This improvement likely stems from the enhanced bonding effect induced by the extracellular polymeric substances (EPSs) released by the microorganisms [42]. These EPSs promote a stronger bond between the concrete matrix and the CaCO_3_ gels, forming a semi-solid colloid, thereby increasing the overall splitting tensile strength [83]. In congruence with these findings, Akhtar et al. [84] demonstrated that concrete incorporating recycled concrete coarse aggregates and cemented bacteria exhibited an approximate 31% increase in splitting tensile strength compared to the control group after 28 days of curing.

**Figure 13:**
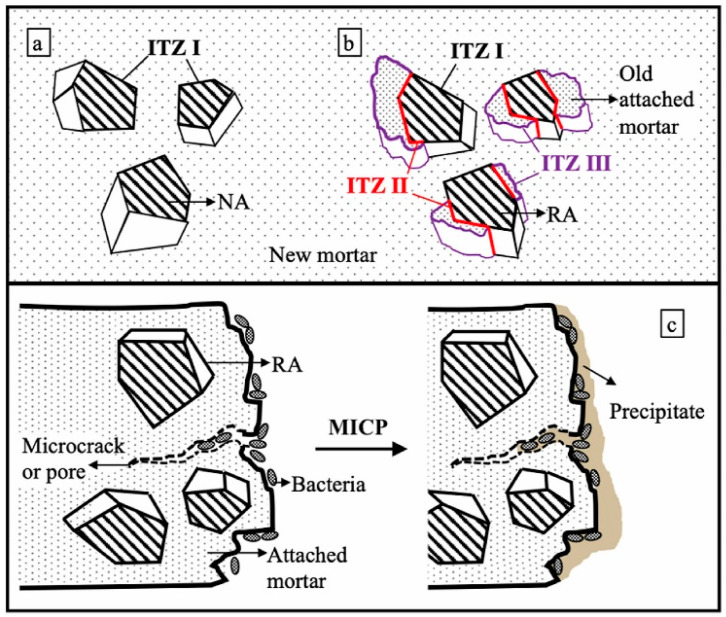
(**a**) ITZ I: interface between NA and newly formed mortar; (**b**) ITZ II: interface between RA and its adherent old mortar; ITZ III: interface between the attached old mortar (from RA) and the newly mixed mortar; (**c**) MICP [87].

The incorporation of RA into concrete results in the formation of three ITZs, as illustrated in Figure 13a,b [85]. These ITZs constitute inherent weak zones within the cementitious matrix, directly contributing to the diminished performance characteristics observed in RC [86]. However, MICP treatment can mitigate these weaknesses. The generated CaCO_3_ gels exhibits favorable compatibility with the cement paste, leading to improved properties within the ITZ of RC and, consequently, enhanced mechanical properties of the resulting RC, as depicted in Figure 13c [87].

**Figure 14:**
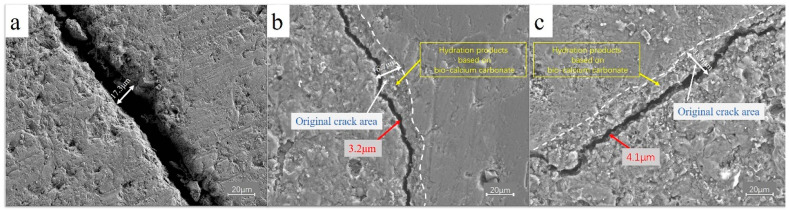
SEM images of concrete ITZ under different MICP treatments: (**a**) control; (**b**) concrete using immersion-treated RA; (**c**) concrete using spray-treated RA [72].

Wu et al. [72] revealed variations in the crack width at the ITZ of concrete prepared with RA treated by different MICP methods, as illustrated in Figure 14. The ITZ crack width decreased from 17.3 μm to 3.2 μm in concrete using immersion-treated RA, and to 4.1 μm with spray-treated aggregates. Microstructural analysis confirmed that the ITZ cracks were effectively filled with biogenic CaCO_3_ gels. These biologically precipitated gel minerals served as nucleation sites for further hydration products, resulting in a denser composite matrix compared to untreated control samples [54,88]. Furthermore, Zhao et al. [55] investigated the microhardness of the ITZ in RC before and after MMGN treatment, reporting values in the range of 23–26 HV for ITZ1 (between NA and new mortar) and ITZ2 (between old and new mortar). Following MMGN treatment, the microhardness of ITZ2 increased from 23.08 HV to 25.91 HV, even exceeding the microhardness between NA and new mortar (25.64 HV). These results showed that MICP modified RA, which reduced its porosity and water absorption, and more free water participated in cement hydration, which improved the ITZ properties of RC and resulted in improved mechanical properties.

In summary, MMGN treatment effectively reduces RA porosity. The resultant bio-CaCO_3_ gel forms chemical bonds (Ca-O-Si) with cement hydration products (C-S-H), fostering a transformation of the C-S-H structure from a planar arrangement to a three-dimensional spatial network. This process strengthens the ITZ within concrete composites.

## 4. Self-Healing Characteristics of MMGN-Enhanced RC

Beyond enhancing the mechanical properties of RC, MICP can introduce bacterial spores and nutrients into the aggregate during concrete production. This process yields a CaCO_3_ gel with biomineralization-induced self-assembly properties. Its inorganic crystalline framework confers structural rigidity while precisely regulating porosity, thereby enabling autonomic restoration of concrete performance [89]. The efficacy of this self-healing mechanism can be comprehensively evaluated based on three key parameters, as follows: crack healing efficiency, assessed through the reduction in crack width and achievement of complete crack closure; mechanical property recovery, quantified by the restoration of strength and stiffness following pre-induced damage; and permeability recovery, determined by the regained waterproofing performance after repair.

### 4.1. Crack-Making Method

**Figure 15:**
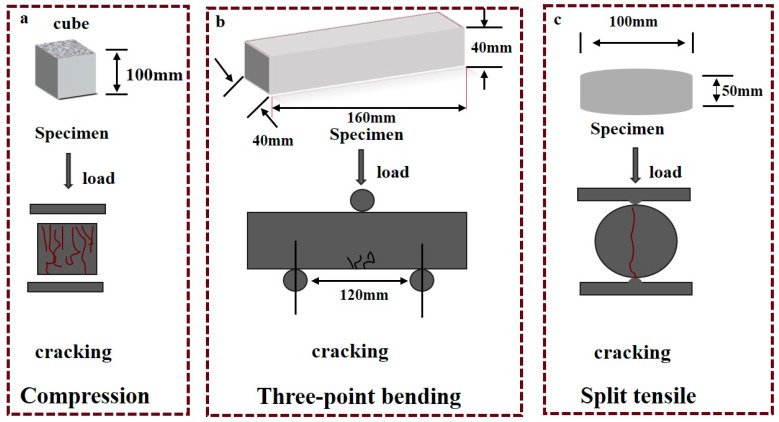
Crack-manufacturing methods for concrete. (**a**) Compression; (**b**) Three-point bending; (**c**) Split tensile.

To evaluate the crack-healing efficacy of MICP in concrete, researchers have employed three-point bending, compressive, and split tensile tests to induce cracking. These diverse crack fabrication methods allow for a comprehensive assessment of different facets of bacterial repair. Three-point bending and compressive tests are primarily utilized to investigate crack width reduction, whereas the split tensile test evaluates not only the healed crack width and area but also the depth of crack repair. In three-point bending, and compressive tests, the applied load typically ranges from 60% to 90% of the maximum load, ceasing upon the initial appearance of cracks on the concrete surface. Figure 15 provides schematic representations of these three crack-fabrication methodologies.

### 4.2. Self-Healing Effects

#### 4.2.1. Crack-Healing Effects and Influencing Factors

During concrete preparation, bacterial spores, often in a dormant state, and necessary nutrients are incorporated within the aggregate. Upon the formation of cracks and fractures in the concrete, these spores are exposed to water and atmospheric oxygen, triggering them into active vegetative cells. These cells then produce CaCO_3_ gels, which contributes to crack remediation. Luo et al. [65] employed MMGN-reinforced RA as a carrier for bacteria, observing that RA afforded enhanced bacterial protection, resulting in increased CaCO_3_ gel precipitation and a self-healing rate of up to 94.5% of the crack width within 56 days. Khushnood et al. [90] utilized RA and natural fine aggregate as bacterial carriers, achieving crack healing up to 1.1 mm, compared to only 0.7 mm in specimens containing only RA. Other researchers have explored auxiliary methods to augment the crack-healing efficacy of MICP in RC. Rais et al. [91], for example, incorporated microsilica and metakaolin into self-healing concrete and, after a 56-day incubation period, achieved complete healing of cracks with a maximum width of 0.63 mm, thereby enhancing the long-term durability of the recycled concrete. Bakr et al. [92] investigated the use of bacterially treated banana fibers and RCA to assess the efficiency of these materials as bacterial spore carriers in self-healing concrete, reporting accelerated fiber matrix regeneration and crack sealing via calcite precipitation, ultimately restoring flexural properties. Xu et al. [93] demonstrated that alkaline activation (via sodium silicate) of RA significantly enhances MICP-mediated crack self-repair, despite inducing a reduction in splitting tensile strength due to RA coating interactions. Table 2 summarizes the reported effectiveness of crack healing under various conditions. It is worth noting that the crack recovery effect using RCA and natural fine aggregate immobilized bacteria is better compared to using only RCA immobilized bacteria. In addition, MMGN-treated RCA-immobilized bacterial spores have shown promising crack repair capabilities.

The initial crack width significantly influences the efficacy of MICP-based repair [95,96]. While Amjad et al. [82] observed successful crack healing across varying widths (0.1, 0.3, 0.4, and 0.6 mm) and reported enhanced MICP efficiency at 0.6 mm compared to 0.4 mm, suggesting that larger fissures may promote microbial metabolism, Rais et al. [91] posited a contrasting perspective. They concluded that excessively wide cracks could facilitate the diffusion of gels precipitates and self-repairing agents into the surrounding water, potentially leading to incomplete crack remediation.

Furthermore, the curing age of RC prior to cracking appears to affect healing efficiency. The superior performance observed in pre-cracking crack healing at 3 days of curing, relative to concrete cured for 7 and 28 days, has been attributed to the early depletion of available nutrients, which may hinder their transport through the cracked region. Concurrently, the higher viability of bacteria at earlier curing stages may contribute to improved crack healing [90].

#### 4.2.2. Mechanical Properties After Self-Healing

While incorporating bacterial spores into aggregate can restore mechanical properties through crack-induced precipitation, variations in microbial species, carrier type, cementation method, and curing environment can influence the efficacy of self-healing in microbial concrete. For instance, Wang et al. [75] employed a strategy where half of the RA was used to encase nutrients and calcium sources, while the other half was used to immobilize bacterial spores. Their study revealed that after pre-destructive treatment, specimens cured for 7 days and allowed to recover for another 7 days exhibited fully restored compressive strength, comparable to specimens cured for 14 days. Furthermore, the extent of compressive strength recovery was shown to vary depending on the age at which cracking occurred. While microbially mediated compressive strength recovery shows significant potential, its effectiveness is highly contingent upon multiple governing parameters. For instance, studies [90] have shown that concretes incorporating RCA and NFA techniques exhibit diminishing self-healing capabilities as the age at which cracking occurs increases. Specifically, compressive strength recovery after 28 days of healing was reported as 85% when cracking occurred at 3 days, decreasing to 82% and 78% for cracking ages of 7 and 28 days, respectively. This trend can be attributed to the increasing alkalinity of the concrete matrix and the progressive densification of its microstructure as curing time advances, which consequently reduces bacterial activity and, by extension, the self-healing potential [76]. Furthermore, research by Amjad et al. [82] indicates that fine aggregate samples incorporating bacteria exhibited a compressive strength recovery of 83.5% after 28 days of healing, whereas coarse aggregate samples with bacteria showed a higher recovery rate of 89.7%. This discrepancy may stem from the extended survival of microorganisms within the larger pore structure of the RCA [91]. Consequently, it is evident that the self-healing performance of microbial concrete is context-dependent; however, the application of MICP generally contributes to an enhancement in compressive strength restoration.

Liu et al. [94] examined the flexural stiffness recovery of reinforced concrete beams subjected to four-point bending, incorporating bacterial spores within RA. Their findings revealed that RC specimens treated with bacterial spore-amended RA exhibited a 12.25% increase in bending stiffness after 28 days of healing in water via oxygenated pumping. This improvement rose to 53.02% after 56 days, suggesting the CaCO_3_ gels produced by bacterial activity possesses significant adhesive properties [97]. These CaCO_3_ gels’ precipitation extended beyond the initial localized area to bridge the entire crack, effectively connecting both sides and enhancing the interfacial continuity and adhesion between particles [98]. Consequently, this process not only facilitated crack closure but also contributed to the restoration of the specimen’s flexural stiffness. However, as summarized in Table 3, which presents a comparative overview of mechanical property recovery in concrete after pre-induced damage, while compressive strength typically recovers to above 80% after self-healing, the recovery of bending stiffness tends to be limited to approximately 50%.

In summary, the MICP technique facilitated the repair of pre-existing cracks in RC and effectively restored the mechanical properties of reinforced concrete elements.

#### 4.2.3. Permeability After Self-Healing

**Figure 16:**
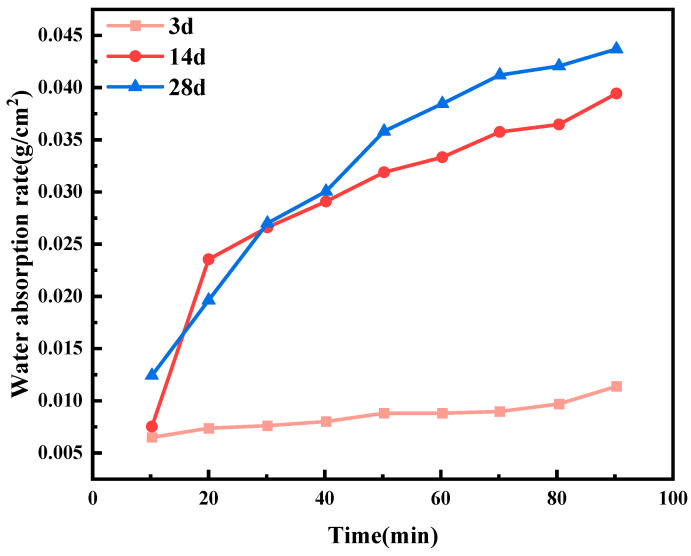
Water absorption of self-healing concrete specimens under varying crack times.

Wang et al. [75] validated the effectiveness of microbially induced mineral deposition in repairing cracks and enhancing concrete durability. After 28 days of alternative wet–dry cycles, microorganism-treated specimens achieved near-complete water impermeability recovery (99.2%). Their findings further suggest that the timing of cracking significantly influences the subsequent recovery of watertightness. Specifically, concrete samples pre-cracked within 3 days exhibited a lower water absorption rate after 28 days of healing compared to samples pre-cracked at 14 and 28 days (Figure 16). This observation indicates that accelerated crack repair stems from enhanced microbial viability and metabolic activity during early-phase colonization, directly enabling more effective restoration of specimen impermeability [80].

In summary, incorporating bacterial spores into recycled concrete effectively facilitated property restoration following pre-cracking. The initial crack width and the timing of pre-cracking significantly influenced the efficacy of crack recovery in the recycled concrete. The CaCO_3_ gels generated through MICP effectively filled the cracks, leading to improved watertightness in the concrete specimens, with earlier cracking ages resulting in superior watertightness restoration. However, research on chloride ion penetration in repaired recycled concrete remains limited, necessitating further investigation into the long-term performance and durability characteristics of repaired concrete structures.

## 5. Environmental and Economic Benefits of MMGN-Enhanced RC

MMGN-enhanced RC offers significant environmental advantages. Primarily, the MICP process facilitates carbon dioxide sequestration from terrestrial sources, buildings, or the atmosphere. This process, mediated by carbonic anhydrase, generates carbonate ions [99] which subsequently precipitate as carbonate minerals on the surface of bacterial cells. These minerals exhibit long-term stability and enable efficient carbon dioxide storage [100]. For instance, Wang et al. [87] demonstrated that *Bacillus mucilaginosus* (CICC 23640) enhances RA by accelerating carbon dioxide dissolution and promoting carbonate ion production, thereby facilitating CO_2_ absorption. Furthermore, the utilization of RA, compared to NA, also reduces carbon emissions. The production of 1 ton of NA releases 0.046 tons of CO_2_, while the production of 1 ton of RA emits only 0.0024 tons of CO_2_ [101]. A study further indicated that incorporating 50% RA in road construction projects, as a substitute for NA, can decrease greenhouse gas emissions by approximately 23.0% [102].

The economic advantages of MMGN-enhanced RC are further amplified by the incorporation of RA. The utilization of RA significantly diminishes raw material expenses; the long-term cost of producing one ton of coarse recycled concrete aggregate is approximately 40% less than that of producing coarse NA [103]. Furthermore, Feng et al. [104] conducted a life cycle assessment of various technical enhancement processes for RA and identified MICP as the most cost-effective method, but this depends on all the raw materials being obtained from nature. However, the initial cultivation of microorganisms, a critical step in MICP-based enhancement, represents a substantial cost burden that is difficult to alleviate. Consequently, in the more complex environmental conditions characteristic of real field applications, the cost-effectiveness of MICP warrants further investigation to provide robust scientific guidance for practical engineering implementations.

## 6. Limitations and Outlook of MMGN-Enhanced RC

Although microbial mineral gel-enhanced RC can restore the compressive strength and crack resistance of RC and bring some environmental benefits, there are still several limitations in the current field of research, including the following:

(1) Current research on MMGN-enhanced RC mostly focuses on its short-term properties. As a result, future studies should prioritize investigating the long-term durability of MMGN-enhanced RC. Furthermore, when applying this technology to reinforced concrete structures in marine engineering, special attention should be paid to studying certain properties, such as its chloride permeability. This will provide a more solid foundation for the application of MICP concrete in practical engineering projects.

(2) Although existing studies have investigated bacterial spore immobilization using RCA or combinations of RCA and NFA, research on employing RFA for bacterial spore immobilization to enhance the distribution uniformity of self-healing agents remains relatively limited. Consequently, future research should focus on simultaneous immobilization of bacterial spores using both RCA and RFA, leveraging their synergistic effects to improve the distribution efficiency of self-healing agents. This approach will enable a comprehensive evaluation of their impact on concrete performance. Such methodology will provide a holistic understanding of the potential advantages and inherent limitations of using RA in bio-concrete applications, thereby further reducing concrete production costs.

(3) Current research on MICP technology remains predominantly confined to laboratory settings with limited utilization of RA. While these controlled environments require modest microbial and nutritional resources, practical applications demand substantially larger material volumes. Consequently, to enable widespread adoption of microbial concrete products, future research must prioritize translating laboratory findings to real-world projects by developing cost-effective strategies for microbial cultivation and nutrient supply, alongside establishing standardized engineering protocols for microbial consortia. This is critical to mitigate and potentially reverse the impacts of such materials on pressing environmental concerns, like climate change and soil contamination. Furthermore, advancements in production technology and field implementation strategies remain imperative. Addressing inherent challenges in large-scale manufacturing, transportation, and deployment is essential to fully realize the potential of microbial concrete.

(4) Existing studies predominantly examine MICP at a laboratory scale. The efficacy of bacterial activity, crack characteristics, and long-term self-healing performance may differ considerably in the complex environment of in situ RC structures, especially when the concrete ages, multiple cracks appear, or it is subjected to various sustained loads. Consequently, future investigations should prioritize the application of MICP in RC containing steel reinforcement under realistic environmental conditions to better assess its long-term durability and effectiveness.

## 7. Conclusions

MICP offers a dual benefit, simultaneously enhancing RC and imparting it self-repairing capabilities, leading to enhanced durability compared to conventional self-healing methods. Key conclusions drawn from this review are summarized as follows:

(1) The microorganisms examined, particularly urease-producing bacteria, exhibit optimal activity within the alkaline environment at mesophilic temperatures (20–37 °C). Careful control of reactant dosing is crucial, as moderated precipitation kinetics result in superior crystalline structures and interfacial bonding compared to rapid deposition.

(2) The observed enhancement of mechanical properties of RC through MICP is primarily attributed to the following three synergistic mechanisms: (a) a pore-filling effect, wherein biogenic gels precipitates effectively seal macro-defects within RA; (b) interfacial optimization achieved through the excellent chemical compatibility between CaCO_3_ gels and the cement matrix; and (c) a nucleation effect, accelerating the formation of C-S-H and forming new chemical bonds (Ca-O-Si) with calcium carbonate gel, thereby densifying the ITZ.

(3) RA is an excellent self-healing concrete microbial carrier. RC made from RA loaded with bacterial spores exhibits excellent crack closure, mechanical performance recovery (85–95%), and durability recovery.

(4) Although MMGN-enhanced RC demonstrates significant environmental and economic benefits, certain limitations persist. These include the need for long-term performance studies; research on utilizing RFA to improve the dispersion uniformity of self-healing agents; and further investigation into its long-term durability and effectiveness in realistic complex environments where reinforced structures are present.

## Figures and Tables

**Table 1:** The enhancement of compressive strength of MMGN-enhanced RC.

Bacteria	Type of Concrete	w/c	Bacterial Concentration	Percentage Increase in Compressive Strength (28 Days) Relative to Untreated RC	Reference
*B. pseudofirmus*	a	0.5/0.35	1 × 10^8^ cells/mL	35.5%/20.9%	[7]
*B. subtilis*	a	0.55	-	16.7%	[19]
*B. cohnii*	b	0.40	OD_600_ = 1	10.8%	[22]
*B. megaterium*	a	0.4	3 × 10^6^ cells/mL	27.8%	[23]
*B. pasteurii*	a	0.49	-	19.54%	[24]
*Pseudomonas* Sp.	b	0.45	1 × 10^9^ CFU/mL	25.7%	[32]
*B. pasteurii*	a	0.49	4.5 × 10^8^ cells/mL	15.04%	[54]
*S. pasteurii*	a	0.5	1.85 × 10^8^ cells/mL	14.3%	[53]
*B. mucilaginosus* and *B. subtilis*	a	0.5	2 × 10^8^ cells/mL	19.02%	[35]
*S. pasterurii*	b	0.49	-	16.6%	[65]
	b	0.5	4.73 × 10^8^ cells/mL	−14%	[75]
	a	0.55	1 × 10^8^ cells/mL	6.2%	[55]
	a	0.5	-	5.8%	[45]
*B. subtilis*	b	0.5	1 × 10^9^ cells/mL	30%	[78]
*B. mucilaginosus* and *B. subtilis*	a	0.5	*B. mucilaginosus*:1.2 × 10^8^ cells/mL; *B. subtilis*: 2.4 × 10^8^ cells/mL	11.1%	[79]
*B. sphaericus*	b	0.5	1 × 10^5^ cells/mL	−44.2%	[80]

Note: The types of concrete presented in this table are illustrated in Figure 9.

**Table 2:** The effect of crack healing under different conditions.

Method of Crack Creation	Bacterial Spore Sequestration	Healing Condition	Healing Effect	Reference
Splitting resistance	MMGN-treated RCA-fixed bacterial spores	-	For a crack with a width of approximately 0.4 mm which healed in 56 d, the repair rate of the crack width was 94.5%	[65]
	Half of the RCA is fixed with bacterial spores, while the other half is fixed with substrate and calcium sources	Specimens were immersed in water for 16 h and exposed to air for 8 h	At a crack width of 0.6 mm, the average healing rate and crack area healing rate were 71% and 84%, respectively, with a crack healing depth of 17.8 mm and approximately 100% recovery of water tightness	[75]
Pressure-resistant methods	RCA and 50% natural fine aggregate (NFA)	Underwater curing	Successfully healed a maximum width of 1.1 mm	[90]
	Recycled coarse brick aggregate	Underwater curing	0.6- mm crack healing	[82]
	RCA	Underwater curing	Cracks of 0.2–0.8 mm basically healed in 28 d, and the crack-healing rate was related to crack width and crack age	[91]
Three-point flexural	RCA	Oxygenated by an oxygenating pump with the specimen completely submerged in water	At 28 d, a 0.25 mm crack was healed	[94]
	RCA	Subject the samples to a 24 h water immersion followed by an 8 h room temperature drying period, repeating this cycle continuously	Following a 28 d healing period, cracks initially narrower than 300 μm exhibited near-complete closure	[80]

**Table 3:** Recovery effect of mechanical properties under different loading modes.

Bacteria Type	Bacterial Spore Sequestration	Bacterial Concentration	Mechanical Property Recovery Effect	Reference
*S. pasteurii*	RCA	4.73 × 10^9^ cells per cubic meter of concrete present	99.7% recovery of compressive strength	[75]
*Lysinibacillus boronitolerans*	RCA	Biomimetic agent (3 × 10^6^ cells/mL)	89.7% restoration of compressive strength	[82]
*B. pumilus*	RCA	2.23 × 10^9^ cells/mL cubic meter of concrete	79% restoration of compressive strength	[84]
*B. subtilis*	RCA and 50%NFA	1.9 × 10^7^ cells/cubic centimeter of concrete	85% compressive strength recovery at 3 days, 82% recovery at 7 days, and 78% recovery at 28 days of age at cracking	[90]
*B. pasteurii*	RCA	The concentration of bacterial spores was 2.8 × 10^9^ cells/ml	The bending stiffness healing rate was 12.25% at 28 days and 53.02% at 56 days of healing	[94]

## Data Availability

No new data were created or analyzed in this study.
